# Masked transposition priming effects are observed in Korean in the same–different task

**DOI:** 10.1177/1747021821997336

**Published:** 2021-03-15

**Authors:** Chang H Lee, Clare Lally, Kathleen Rastle

**Affiliations:** 1 Department of Psychology, Sogang University, Seoul, Korea; 2 Department of Psychology, Royal Holloway, University of London, Egham, UK

**Keywords:** Orthographic processing, masked priming, position coding, cross-linguistic, Korean Hangul, transposition priming

## Abstract

Research suggests that readers of Korean Hangul demonstrate precise orthographic coding. In contrast to findings from many other languages, the identification of Hangul words is not speeded by prior masked presentation of transposition primes relative to substitution primes. The present studies asked whether evidence for precise orthographic coding is also observed in the same–different task—a task claimed to reflect pre-lexical orthographic representations. Experiments tested whether masked transposed-letter (Experiment 1) or transposed-syllable-block (Experiment 2) primes facilitate judgements about whether a target matches a reference stimulus. In contrast to previous results using lexical decision, significant transposition effects were observed in both cases. These findings add weight to the proposition that apparent differences across writing systems in the precision of orthographic coding may reflect demands of the word identification process rather than properties of orthographic representations themselves.

The recognition of printed words requires analysis of letter identity and letter position. Analysis of letter identity allows us to distinguish visually similar words like *step* and *stop*, while the analysis of letter position allows us to distinguish words like s*tep* and *pest* comprising the same letters in different positions. The past 15 years has seen substantial interest in the coding of letter position in skilled reading.

The first generation of computational models, such as the dual-route cascaded model (DRC; Coltheart et al., 2001) and the multiple read-out model (MROM; Grainger & Jacobs, 1996), handled letter position through slot-based coding. In slot-based coding, printed words are represented through banks of letters within fixed positions. The consequence of this representational scheme is that printed words comprising the same letters in different positions (e.g., P_1_E_2_S_3_T_4_ vs S_1_T_2_E_3_P_4_) have no more similarity than words comprising totally different letters (e.g., P_1_E_2_S_3_T_4_ vs R_1_O_2_A_3_R_4_). However, substantial research conducted since the release of these models indicates that this is not a good description of orthographic processing in skilled readers.

This research has generally shown that stimuli with letter transpositions such as *jugde* show greater perceptual similarity with their base words (e.g., *judge*) than do stimuli with replaced letters (e.g., *jupte*; Perea & Lupker, 2003). Studies of unprimed lexical decision have shown that transposed stimuli are more difficult to classify as nonwords than non-transposed controls (e.g., Lupker et al., 2008). Similarly, studies of masked priming have shown a transposed-letter priming benefit relative to substitution controls (e.g., sevrice-SERVICE vs. sedlice-SERVICE; Schoonbaert & Grainger, 2004). These findings have motivated a number of alternative conceptualisations of orthographic processing that predict some degree of uncertainty in the perception of letter position (e.g., Davis, 2010; Gomez et al., 2008; Grainger & Whitney, 2004; Norris & Kinoshita, 2012b).

Although transposed letter phenomena have been reported in many languages, recent findings have yielded some exceptions. Velan and Frost (2007, 2009, 2011) published a series of studies demonstrating that Hebrew readers appear to be much less tolerant to letter transpositions than would be expected in other languages such as English. They made the case that the extreme rigidity of Hebrew letter position coding derives from the importance of the tri-consonantal root structure in word identification. Because Hebrew tri-consonantal roots are built from just 22 letters, roots often consist of the same letters in different positions. Flexibility in position coding would frequently lead to root misidentifications (Frost, 2012). Similar findings have since been reported for Arabic, another Semitic language with a non-concatenative, tri-consonantal root morphology, in which root identification depends critically on position information (Boudelaa et al., 2019; Perea et al., 2010).

These original claims regarding position rigidity focused on aspects of Semitic morphology (i.e., the importance of the root in word identification). However, Lerner et al. (2014) operationalised this proposal more generally in terms of the prevalence of anagrams in known orthographic forms. They developed a series of computational models demonstrating that reliance on positional information emerges more strongly when training sets have a high proportion of anagrams. Laboratory experiments in which adults learned to read in artificial writing systems designed to have many anagrams or none were consistent with this pattern (Lally et al., 2020). These studies suggest that the rigidity or flexibility of position coding may not be tied to specific morphological structures but may instead be a consequence of the orthographic density of a writing system (Frost, 2012).

The proposal that the rigidity of position coding may vary as a function of the properties of a writing system is supported by research on visual word recognition in Korean Hangul. Korean Hangul is an alpha-syllabic writing system in which words are written in syllable blocks, with syllable blocks separated by a physical gap. Syllables comprise two, three, or four letters in a fixed structure (CV, CVC, or CVCC) and physical location within the block. Previous research has highlighted two interrelated aspects of the writing system that might be expected to yield rigid position coding. The first is that the location of the onset, vowel and coda within the syllable block is fixed and unambiguous. This means that letters may be assigned to these sub-syllabic positions very rapidly, and for this reason we might not expect to see any evidence of uncertainty in position coding, such as transposed-letter effects (Lee & Taft, 2009, 2011). The second is that because of this fixed syllable block structure, the orthographic space is very dense, with a high proportion of anagrams. Transpositions within syllable blocks frequently yield another syllable, and transpositions of whole syllables frequently yield another word (Rastle et al., 2019). On the logic of Lerner et al. (2014), this property makes Hangul reading likely to exhibit rigid position coding.

This hypothesis that Hangul should exhibit rigid position coding is supported by evidence from both unprimed lexical decision and masked priming of lexical decision. Lee and Taft (2009) created disyllabic English and Korean stimuli with transpositions across the first and second syllables. These transpositions involved swapping onsets with onsets, codas with codas, and codas with onsets across the syllable block boundary. They measured whether these items were harder to reject in lexical decision than substitution controls. Although English readers showed a substantial transposed-letter disadvantage, this was not observed for Hangul readers. Lee and Taft (2011) went on to show an absence of transposed-letter effects on unprimed lexical decision when the onset and coda were swapped within a syllable. More recently, Rastle et al. (2019) conducted a set of high-powered masked priming experiments, investigating the impact of transposition primes on recognition of Hangul words relative to substitution controls. In their first two experiments, they tested whether primes with onset-coda transpositions within syllables would facilitate recognition of their base words. Despite observing robust identity priming effects, they found no benefit from these primes on recognition of monosyllabic or disyllabic target words. In a third experiment with Hangul readers, they studied the impact of masked primes that transposed the syllable blocks of disyllabic targets. Despite the fact that these primes shared exactly the same syllable blocks as targets, and despite observing robust identity priming effects, they again found no benefit of these primes relative to a substitution condition in which both syllable blocks differed from those comprising the targets. This final experiment also included a condition in which primes and targets shared a single syllable block in the same or different position; no priming was observed from these primes either. Bayesian statistics provided evidence for the strength of these null effects.

Rastle et al. (2019) argued that these results suggest a very high degree of rigidity in orthographic coding in Hangul reading. They argued that while the absence of transposed letter effects could be understood through Lee and Taft’s (2009, 2011) proposal regarding rapid assignment of letters to positions within the syllable block, it was more difficult to understand the absence of priming for transposed syllable blocks within this account. They therefore favoured an explanation in terms of the orthographic density of the Hangul writing system. Overall, the data of Rastle et al. (2019) appear consistent with the arguments of Frost (2012; also Lerner et al., 2014): position flexibility or rigidity in orthographic representations is not “universal” across writing systems but is a learned consequence of the extent to which position information is required in word identification.

However, one prominent challenge to Frost’s (2012) position suggests that uncertainty in the perception of letter order *is* universal across writing systems, and that any variation observed across writing systems reflects aspects of the recognition process (e.g., Gomez & Silins, 2012; Norris & Kinoshita, 2012a). Norris and Kinoshita (2012a) sum up this position in the title of their response to Frost (2012): “Orthographic processing is universal; it’s what you do with it that’s different.” Kinoshita et al. (2012) provided support for this hypothesis by demonstrating that a transposed-letter priming effect *is* observed in Hebrew when using the same–different task (see also Boudelaa et al., 2019, for analogous findings in Arabic). In the same–different task, a masked prime is presented between a reference stimulus and a target, and participants are required simply to decide whether the target is the same or different from a reference stimulus.

There have been two related claims seeking to explain why transposed-letter priming for Hebrew (Kinoshita et al., 2012) and Arabic (Boudelaa et al., 2019) is observed in the same–different task but not in the lexical decision task. The first claim is that the same–different task reflects pre-lexical orthographic representations, and that precise position coding in these writing systems arises during the process of lexical identification (e.g., Kinoshita et al., 2012). The claim that the same–different task reflects pre-lexical orthographic representations is based on the fact that masked priming effects for nonwords are observed in this task (in contrast to masked priming of lexical decision; Kinoshita & Norris, 2009; Norris & Kinoshita, 2008). The second claim is that responding in the same–different and lexical decision tasks requires different degrees of evidence. Responding in the same–different task requires sufficient evidence to determine whether a target is the same as a reference stimulus, not about whether the target is a word (Norris & Kinoshita, 2008). Because the target stimulus is not being assessed against the whole lexicon, distributional or structural characteristics of the writing system are unlikely to influence responding (Boudelaa et al., 2019). Conversely, when the task is to identify whether a stimulus is a known word, substantial evidence regarding letter identity and position is required in Hebrew and Arabic because of the risk of activating the wrong tri-consonantal root (Norris & Kinoshita, 2012a). The same degree of evidence is not required in English lexical decision due to its low orthographic density; according to Norris and Kinoshita (2012a), “English readers can tolerate more slop in the system.”

This body of work now appears to suggest that letter position coding in Hebrew (Kinoshita et al., 2012) and Arabic (Boudelaa et al., 2019) is characterised by a degree of perceptual uncertainty, and that the apparent rigidity of position coding in these writing systems arises in the process of word identification. The present research was conducted to determine whether this pattern is restricted to Semitic writing systems (in which lexical identification involves analysis of the tri-consonantal root), or whether it might also be observed in Korean Hangul. We therefore conducted two experiments testing whether the null effects of transposed-letter and transposed-syllable-block priming in Korean Hangul observed by Rastle et al. (2019) also emerge when using the same–different task. If these priming effects remain null in this task, then our findings would depart from those of Kinoshita et al. (2012) and Boudelaa et al. (2019), and we would conclude that the rigid position coding observed previously by Rastle et al. (2019) characterises orthographic representations themselves (rather than what one does with them; Norris & Kinoshita, 2012a). Conversely, if transposed-letter and transposed-syllable-block priming effects do emerge in this task, then that would give weight to the position of Kinoshita et al. (2012; also Boudelaa et al., 2019) that orthographic representations are characterised by position flexibility, and that position rigidity emerges in some writing systems as a consequence of processes arising during word identification.

## Experiment 1

Experiment 1 investigated masked transposed-letter priming of disyllabic Hangul words and nonwords using the same–different task. The design of the same–different task closely followed Kinoshita et al. (2012), and we anticipated that any priming effects should be observed on both word and nonword targets (Kinoshita & Norris, 2009; Norris & Kinoshita, 2008). Prime conditions were modelled closely on Rastle et al. (2019, Experiment 3): identity, onset-coda transposition in the first syllable, onset-coda transposition in the second syllable, and matched substitution controls for the latter two conditions. Rastle et al. (2019) reported significant identity priming (47 ms priming) but null transposed letter priming (1 ms priming) in the masked lexical decision task.

### Method

#### Participants

Seventy-two undergraduate students (54 females) at Sogang University participated in the experiment for course credit. Participants were native Korean speakers who reported having normal or corrected-to-normal vision and no language impairments. The ages ranged from 19 to 26 years (*M* = 21.11, *SD* = 1.72).

#### Materials

A total of 160 disyllabic words with a CVC–CVC structure were selected as targets from the Korean Word Database (frequency = 1,238/15 million, *SD* = 1,394; National Institute for the Korean Language, 2001). These targets were matched to 160 nonword targets with the same structure. Nonwords were constructed by changing one or two letters of a word to make a legal string. Half of the words and half of the nonwords were selected for the *same* and *different* response groups.

For the word stimuli, the *same* and *different* target words were closely matched on neighbourhood size (*M* = 2.89 [*SD* = 2.26], *M* = 3.10 [*SD* = 2.47], respectively; National Institute of the Korean Language, 2001). The *same* and *different* nonword targets were also closely matched on this factor (*M* = 2.33[*SD* = 2.71], *M* = 2.45 [*SD* = 2.57], respectively). Reference words were identical to targets for the *same* response, and did not share any syllables with targets for the different response. For the *different* response, reference words were matched to targets on neighbourhood size (*M* = 2.83[*SD* = 2.43] for word targets; *M* = 2.36[*SD* = 2.55] for nonword targets). Reference stimuli were words for word targets and nonwords for nonword targets.

Primes appeared between the reference stimuli and the targets. Five types of prime were created for each target word and nonword: (a) an *identity* prime (ID; for example, 건물 – 건물); (b) a *transposed letter prime* involving disruption to the first syllable (TL_first; for example, 넉물 – 건물); (c) a *replaced letter prime* involving disruption to the first syllable (RL_first; for example, 헝물 – 건물); (d) a *transposed letter prime* involving disruption to the second syllable (TL_second; for example, 건룸 – 건물); and (e) a *replaced letter prime* involving disruption to the second syllable (RL_second; for example, 건준 – 건물). TL primes were constructed by transposing the target’s onset and coda consonants, while RL primes were constructed by replacing them with different letters. Primes in the four non-identity conditions were all nonwords. The neighbourhood size of the four non-identity conditions was closely matched (for “word” target primes, *M* = 0.82 [*SD* = 1.34], *M* = 0.88 [*SD* = 0.99], *M* = 0.86 [*SD* = 1.29], *M* = 0.86 [*SD* = 0.88], respectively; for “nonword” target primes, *M* = 0.82 [*SD* = 1.74], *M* = 0.81 [*SD* = 1.35], *M* = 0.80 [*SD* = 1.61], *M* = 0.81 [*SD* = 1.12], respectively).

The assignment of primes to targets was counterbalanced across participants such that all participants received all prime conditions but were exposed to each target only once. This counterbalancing variable is termed “List” in the analyses.

#### Procedure

Participants were tested individually in a dark and quiet room and were seated approximately 60 cm in front of an 18-in LCD monitor. They were told that a pair of letter strings would be presented, and they were instructed to determine whether the pair of letter strings matched. Participants recorded their decisions on a two-button response box, with buttons labelled “+” as *same* response and “−” as *different* response. Response times (RTs) and error rates were recorded using DMDX software (Forster & Forster, 2003).

Each participant received a total of 320 experimental trials. The test trials were presented as two blocks of 160 trials each, with a self-paced break. These experimental trials were preceded by 20 practice trials. In each block, half of the trials required a *same* response and the other half required a *different* response. Furthermore, the five priming conditions were represented equally within each block, with items presented in a different random order for each participant. Participants were not told about the existence of the primes.

Each trial consisted of a sequence of three events: (a) a reference item above a forward mask consisting of four hash signs for 1,000 ms, and then the reference item disappeared, (b) the forward mask was immediately replaced by a prime stimulus for 50 ms (based on the monitor’s refresh rate of 16.67 ms of 3 ticks), and (c) the target stimulus for 2,000 ms. The inter-trial interval was 1 s. Font size and style differed across primes and targets to reduce perceptual overlap between them. Specifically, primes were 12-pt. font size with *dokum* style Korean letter, and targets were 15-pt. font size with *batang* style Korean letter. These display parameters were identical to those used by Rastle et al. (2019). Participants were asked to respond as quickly and accurately as possible for each trial; no feedback was given.

### Results

Data cleaning and analysis followed Rastle et al. (2019) as closely as possible. Data were cleaned based on inspection of the participant, item, and data-point distributions for each experiment separately. This procedure was applied prior to the calculation of condition means. These procedures led to the exclusion of six data points less than 100 ms or greater than 1,750 ms. We report analyses only for “same” trials, as only “same” trials show (and are predicted to show) masked priming effects (Kinoshita et al., 2012; Norris & Kinoshita, 2008). Response time (RT) and accuracy data are available in Table 1.

**Table 1.** Mean reaction time (RTs, in milliseconds) and error rates (proportions) in Experiment 1.

Response type/prime condition	Target type (Word Status)			
	Word		Nonword	
	RT *M*	% error Rate	RT *M*	% Error Rate
“Same” response				
Identity	435 (74)	1.9 (3.9)	459 (76)	3.6 (4.9)
TL_First	483 (69)	3.7 (5.3)	495 (73)	5.0 (7.0)
RL_First	489 (79)	5.5 (6.9)	504 (72)	5.8 (7.4)
TL_Second	461 (80)	2.6 (4.2)	489 (79)	5.6 (6.4)
RL_Second	472 (72)	3.2 (5.1)	492 (81)	4.1 (5.9)
“Different” response				
Identity	500 (71)	1.7 (3.1)	507 (82)	2.2 (4.2)
TL_First	500 (83)	2.4 (4.1)	503 (69)	2.3 (3.7)
RL_First	499 (76)	1.4 (3.0)	504 (79)	1.8 (3.3)
TL_Second	501 (82)	2.5 (3.9)	503 (72)	1.4 (3.0)
RL_Second	507 (75)	2.5 (4.2)	501 (75)	2.1 (3.4)

Standard deviations in parenthesis; TL_first = transposed letter of the first syllable; RL_first = replaced letter of the first syllable; TL_second = transposed letter of the second syllable; RL_second = replaced letter of the second syllable.

The RT data were analysed using generalised linear mixed-effects models with the lme4 package (Version 1.1-12, Bates et al., 2015) in R (Version 3.3.1; R Core Team, 2016). The RT data were analysed using two generalised linear mixed-effects models. For the initial analysis, we investigated fixed effects of Condition, Word Status, and List and compared all conditions using the identity condition as a baseline. For the first model, the maximal structure was defined as: lmer (RT ~ Condition × Word Status + List + (1|Subject) + (1|Item)). Each random effect, fixed effect, and interaction term was added to the model one at a time, and likelihood ratio tests (LRTs) determined whether including each term improved the fit of the model. The fit of the model significantly improved when the fixed effect of Condition (*LRT*: χ^2^(4) = 154.55, *p* < .001), Word Status (*LRT*: χ^2^(1) = 94.25, *p* < .001), and the interaction term Condition × Word Status (*LRT*: χ^2^(4) = 12.92, *p* < .05) were included. However, the model fit did not improve when the fixed effect of List was included (*LRT*: χ^2^(4) = 8.96, *p* = .062), indicating that there was no main effect of List. Therefore, the final optimal model was lmer (RT ~ Condition × Word Status+(1|Subject)+ (1|Item)). The intercept represents performance in the Identity condition; all other estimates are relative to this value. There was a main effect of Condition, as all other conditions yielded longer RTs compared with the Identity condition (Intercept: β = 431.21, *SE* = 8.08, *Z* = 53.39, *p* < .001, TL_first: β = 36.20, *SE* = 4.74, *Z* = 7.64, *p* < .001, RL_first: β = 48.36, *SE* = 4.74, *Z* = 10.20, *p* < .001, TL_second: β = 21.69, *SE* = 4.72, *Z* = 4.60, *p* < .001, RL_second: β = 33.07, *SE* = 4.73, *Z* = 6.99, *p* < .001). There was also a main effect of Word Status, as RTs were shorter for words compared with nonwords (β = −37.17, *SE* = 4.99, *Z* = −7.44, *p* < .001). Finally, there was an interaction between Condition and Word Status, as the difference between words and nonwords was smaller in RL_first compared with the Identity condition (β = −17.43, *SE* = 6.76, *Z* = −2.58, *p* < .001).

For the second analysis, we removed the identity condition from the dataset and compared the factors of Word Status, TL/RL, Syllable, and List. The maximal structure was defined as lmer (RT ~ Word Status × TLRL × Syllable + List + (1|Subject) + (1|Item)). As before, LRTs determined whether each effect improved the fit of the model. The fit of the model improved significantly when the fixed effect of Word Status (*LRT*: χ^2^(1) = 73.88, *p* < .001), TLRL *(LRT*: χ^2^(1) = 10.99, *p* < .001), and Syllable (*LRT*: χ^2^(1) = 13.72, *p* < .001), as well as the interaction term Word Status × TLRL × Syllable (*LRT*: χ^2^(3) = 10.16, *p* = < .05), were included. However, the model fit did not improve when the fixed effect of List was included (*LRT*: χ^2^(4) = 7.98, *p* = .092), indicating that there was no main effect of List. Therefore, the final optimal model was lmer (RT ~ Word Status × TLRL × Syllable + (1|Subject) + (1|Item)). There was a main effect of Word Status (β = 19.69, *SE* = 5.04, *Z* =3.91, *p* < .001), as RTs were shorter for words compared with nonwords. There was also a main effect of TLRL (β = −12.14, *SE* = 4.72, *Z* = −2.57, *p* < .05), as transposed letter primes (TL_first, TL_second) yielded shorter RTs compared with replaced letter primes (RL_first, RL_second). Finally, there was a main effect of Syllable (β = −15.23, *SE* = 4.72, *Z* = −3.23, *p* < .01), as RTs were shorter for second syllable manipulations (TL_second, RL_second) compared with first syllable manipulations (TL_first, RL_first). Although including the interaction term improved the fit of the model, none of the interactions reached significance.

Error rates were analysed using logistic generalised linear mixed models due to the binomial nature of the data. The maximal structure for the first model was defined as glmer (Error Rate ~ Condition × Word Status + List + (1|Subject) + (1|Item), family = binomial). LRTs indicated that Condition (*LRT*: χ^2^(4) = 19.83, *p* < .001), and Word Status (*LRT*: χ^2^(1) = 32.12, *p* < .001) improved the fit of the model; however, List (*LRT*: χ^2^(4) = 2.19, *p* = .701) and the interaction term Condition X Word Status (*LRT*: χ^2^(4) = 6.69, *p* = .153) did not. This result indicated that there was no main effect of List and no interaction between fixed effects. Therefore, the final optimal model was glmer (Error Rate ~ Condition + Word Status + (1|Subject) + (1|Item), family = binomial). As with the RT data, the intercept represents performance in the Identity condition; all other estimates are relative to this value. There was a main effect of Condition, as error rates were lower in the Identity condition (Intercept: β = −3.77, *OR* = 0.02, *SE* = 0.15, *Z* = −25.57, *p* < .001) compared with the TL_first (β = 0.44, *OR* = 1.56, *SE* = 0.14, *Z* = 3.10, *p* < .01), and RL_first (β = 0.58, *OR* = 1.79, *SE* = 0.14, *Z* = 4.16, *p* < .001) conditions. However, there was no significant difference in error rates between the Identity and TL_second (β = 0.24, *OR* = 1.27, *SE* = 0.15, *Z* = 1.60, *p* = .109) or RL_second (β = 0.29, *OR* = 1.33, *SE* = 0.15, *Z* = 1.95, *p* = .051) conditions. There was also a main effect of Word Status, as error rates were higher for nonwords compared with words (β = 0.55, *OR* = 1.74, *SE* = 0.09, *Z* = 5.97, *p* < .001).

For the second model, we removed the identity condition from the dataset and compared the factors of Word Status, TL/RL, Syllable, and List. The maximal structure for the model was glmer (Error Rate ~ Word Status × TLRL × Syllable + List + (1|Subject) + (1|Item), family = binomial). LRTs confirmed that the fit of the model was improved by fixed effects of Word Status (*LRT*: χ^2^(1) = 22.17, *p* < .001) and Syllable (*LRT*: χ^2^(1) = 5.97, *p* < .001), but not TLRL(χ^2^(1) = 1.07, *p* = .300) or List (χ^2^(4) = 2.24, *p* = .691. This confirmed that there was no main effect of TLRL or List. The model fit did not improve by including the interaction term Word Status × Syllable, χ^2^(1) = 0.03, *p* = .861. Therefore the optimal model was glmer (Error Rate ~ Word Status + Syllable + (1|Subject) + (1|Item), family = binomial). There was a main effect of Word Status, as error rates were higher for nonwords compared with words (β = 0.50, *OR* = 1.64, *SE* = 0.10, *Z* = 4.91, *p* < .001). There was also a main effect of Syllable, as error rates were lower when the manipulation occurred in the second syllable compared with the first syllable (β = −0.25, *OR* = 0.78, *SE* = 0.09, *Z* = −2.70, *p* < .01).

### Discussion

The results of Experiment 1 showed that “same” judgements for Hangul disyllabic targets in the same–different task were speeded by masked primes with onset-coda transpositions relative to substitution controls. This was a numerically small but highly significant effect. These results depart from our previous work investigating masked transposed-letter priming in visual lexical decision, and indicate that at some level, Hangul transposed-letter primes, and targets have greater perceptual similarity than substitution primes and targets.

One potentially puzzling aspect of the data is the lexicality effect on “same” judgements. Although there was no interaction between lexicality and priming, if this task reflects pre-lexical representations (Kinoshita et al., 2012; Kinoshita & Norris, 2009), then it is not clear why judgements for words should be significantly faster than judgements for nonwords. We will reserve comment on this effect until the “General Discussion,” section having assessed whether it also arises in Experiment 2.

## Experiment 2

Experiment 2 investigated whether processing of disyllabic Hangul words and nonwords in the same–different task is facilitated by masked primes that transpose a disyllabic target’s syllable blocks. Research investigating the precision of orthographic position coding has only rarely considered evidence beyond the level of the letter. Yet, the small body of literature that has done so suggests that transposition effects are obtained on these larger units. Recognition of Japanese Kana is facilitated by transposed mora masked primes (Perea & Pérez, 2009; Witzel et al., 2011). Likewise, masked transposed morpheme effects have been observed in English (Crepaldi et al., 2013) and in Basque (Duñabeitia et al., 2009). Finally, research has shown that transposed morpheme stimuli (Crepaldi et al., 2013) and transposed syllable stimuli (Perea & Carreiras, 2006) slow rejections in English and Spanish lexical decision, respectively (although this latter finding was attributed to an orthographic level of processing).

Hangul syllable blocks are orthographic units that represent phonological syllables, but they derive from Chinese characters and frequently communicate meaningful morphological information (although the meanings of syllable blocks typically differ from the meanings of the whole words in which they reside; for example, the “tail” in “cocktail”). Thus, it could be argued that Hangul syllable blocks are similar to characters and to morphemes; and we might therefore expect to observe syllable block transposition effects like those observed in units larger than the letter in Japanese, English, Spanish, and Basque. Yet, previous research has highlighted the fact that around 30% of disyllabic Hangul words are syllable block transpositions of other words (Rastle et al., 2019). Because accurate information about the position of syllable blocks is vital in Hangul word identification, we might expect to observe rigid position coding of syllable blocks.

The first study to investigate position coding of Hangul syllable blocks reported that rejection latencies in unprimed lexical decision were slowed when nonwords were syllable block transpositions of existing words (Lee et al., 2015). This finding would appear to support flexible coding of Hangul syllable blocks. However, stimuli in this study comprised *four* syllables. Crucially, while around 30% of disyllabic Hangul words are syllable block transpositions of other words (Rastle et al., 2019), less than 0.30% of four-syllable words have this property. Thus, precise information about the position of syllable blocks may be less important in the recognition of these long words. Evidence for rigid coding of syllable blocks comes from the masked priming study reported by Rastle et al. (2019). They reported that recognition of disyllabic Hangul targets is facilitated by masked identity primes (46 ms priming) but not by masked transposed syllable block primes (4 ms priming) relative to an all syllable different control (Rastle et al., 2019). In fact, this study also failed to find priming for a single syllable shared in the *same* position (−1 ms priming). This combination of results led Rastle et al. (2019) to suggest that the precision of Hangul orthographic codes may be a phenomenon that extends beyond position information (see also Kim & Davis, 2002).

The purpose of this new experiment is to investigate masked syllable block priming of disyllabic Hangul words and nonwords in the same–different task. Like Experiment 1, the design of the same–different task closely followed Kinoshita et al. (2012), with any masked priming effects expected on both word and nonword targets. The priming conditions were modelled closely on Rastle et al. (2019, Experiment 5): identity, single syllable block overlap (same position), transposed syllable blocks, and replaced syllable blocks (all syllables different). The key question was whether “same” judgements would be facilitated by prior masked syllable block primes. This result would depart from the findings of Rastle et al. (2019) but would be consistent with the pattern obtained in Experiment 1, and for Semitic languages (Boudelaa et al., 2019; Kinoshita et al., 2012).

### Method

#### Participants

Sixty undergraduate students (35 females) from Sogang University participated in the experiment in exchange for course credit. Participants were native Korean speakers between the ages of 19 and 26 years (*M* = 21.93 years, *SD* = 1.86), with normal or corrected-to-normal vision and no history of reading impairment.

#### Materials

A total of 120 disyllabic words with a CVC–CVC structure were selected as targets from the Korean Word Database (average frequency = 1,150/15 million, *SD* = 2,431; National Institute for the Korean Language, 2001). These targets were matched to 120 nonword targets comprising the same structure. Nonwords were created using new combinations of phonotactically and orthographically legal syllables. The vast majority of syllable blocks in word and nonword targets also carried meaningful morphological information (though in most cases unrelated to the meanings of whole words).

The lists of words and nonwords were divided equally into *same* and *different* response conditions. The *same* and *different* target words were closely matched with respect to orthographic neighbourhood size (*M* = 3.62 [*SD* = 3.08], *M* = 3.58 [*SD* = 3.01], respectively). The *same* and *different* target nonwords were also closely matched with respect to neighbourhood size (*M* = 3.93[*SD* = 3.40], *M* = 3.25 [*SD* = 3.14], respectively). For the *same* response, reference stimuli were the same as target stimuli. For the *different* response, reference stimuli had the same syllable structure as targets, and were matched to targets on neighbourhood size (*M* = 3.92 [*SD* = 2.30] to the target words, *M* = 3.42 [*SD* = 3.67] to the target nonwords, respectively), but did not share any syllables with targets. Reference stimuli were words for the word targets and nonwords for the nonword targets.

Four types of primes were created for each of these targets: (a) an identity prime (ID; for example, 졸업 ->졸업); (b) a syllable prime comprising the same first syllable of the target (1SYL_SAME; for example, 졸견 ->졸업); (c) a transposed syllable prime (TS; for example, 업졸 ->졸업); and (d) a replaced syllable prime comprising totally different syllables (ASD; for example, 묵척 ->졸업). Primes in the non-identity conditions were all nonwords. Neighbourhood size for primes across the three non-identity conditions was closely matched (for “word” target primes, *M* = 2.06 [*SD* = 2.34], *M* = 2.25 [*SD* = 2.42], *M* = 2.24 [*SD* = 2.33], respectively; for “nonword” target primes, *M* = 2.05 [*SD* = 2.66], *M* = 2.08 [*SD* = 2.53], *M* = 2.10 [*SD* = 2.89], respectively). For the *1SYL_SAME* condition, primes were created by changing the second syllable of the corresponding target word. The *ASD* condition contained syllables not used in the targets. These prime conditions were similar to those used in Rastle et al. (2019, Experiment 5).

The assignment of primes to targets was counterbalanced across subjects such that all subjects received all prime conditions but were exposed to each target only once. This counterbalancing variable is termed “List” in the analyses.

#### Procedure

The procedure was the same as in Experiment 1, except that the number of experimental trials was 240 rather than 320.

### Results

Data were cleaned in the same manner as in Experiment 1, following the procedures of Rastle et al. (2019). In this experiment, data from two participants were excluded because of an overall error rate over 15%, and seven individual data points over 1,800 ms were removed. RT data for incorrect responses were also removed. Once again, we report analyses only for “same” trials (following Kinoshita et al., 2012). RT and accuracy data are available in Table 2.

**Table 2.** Mean response times (RTs, in milliseconds) and error rates (percentages) in Experiment 2.

Response type/prime condition	Target type (Word Status)			
	Word		Nonword	
	RT *M* (*SD*)	% Error	RT *M* (*SD*)	% Error
“Same” response				
Identity	425 (72)	2.5 (3.9)	443 (73)	3.6 (5.3)
1SYL_SAME	449 (77)	3.0 (4.7)	463 (82)	3.1 (5.0)
TS	450 (72)	3.8 (5.7)	467 (68)	5.9 (7.4)
ASD	487 (77)	6.3 (6.7)	500 (86)	7.0 (7.2)
“Different” response				
Identity	510 (86)	3.0 (5.5)	499 (79)	2.3 (3.7)
1SYL_SAME	524 (90)	2.0 (3.1)	495 (71)	2.4 (4.1)
TS	509 (81)	2.6 (4.8)	503 (80)	2.2 (3.4)
ASD	509 (79)	2.5 (5.1)	510 (86)	2.2 (3.6)

Standard deviations in parenthesis. 1SYL_SAME: 1st syllable same between the prime and the target; TS: transposed syllable.

The RT data were analysed using generalised linear mixed-effects models with the lme4 package (Version 1.1-12, Bates et al., 2015) in *R* (Version 3.3.1; R Core Team, 2016). The maximal model was defined as: lmer (RT ~ Condition × Word Status + List + (1|Subject) + (1|Item)). Each random effect, fixed effect and interaction term was added to the model one at a time, and LRTs determined whether including each term improved the fit of the model. These comparisons indicated that the fit of the model significantly improved when the fixed effect of Condition (*LRT*: χ^2^(3) = 231.06, *p* < .001) and Word Status (*LRT*: χ^2^(1) = 21.23, *p* < .001) were included. However, the model fit did not improve when the fixed effect of List (*LRT*: χ^2^(3) = 1.95, *p* = .583) or the interaction term were included (*LRT*: χ^2^(3) = 0.76, *p* = .858). This result indicated that there was no main effect of List and no interaction between Condition and Word Status. Therefore, the final optimal model was lmer (RT ~ Condition + Word Status + (1|Subject) + (1|Item)). The intercept represents performance in the ASD word condition; all other estimates are relative to this value. The results showed a main effect of Condition, as all other conditions yielded shorter RTs compared with ASD (Intercept: β = 485.35, *SE* *=* 9.71, *Z* =49.99, *p* < .001, Identity: β = −59.06, *SE* *=* 3.89, *Z* = −15.19, *p* < .001, 1SYL_SAME: β = −36.91, *SE* = 3.89, *Z* =−9.49, *p* < .001, TS: β = −34.59, *SE* 3.91, *Z* = −8.86, *p* < .001). There was also a main effect of Word Status, as RTs were shorter for words compared with nonwords (β = –15.71, *SE* *=* 3.27, *Z* = −4.80, *p* < .001).

Error rates were analysed using logistic generalised linear mixed models due to the binomial nature of the data. The maximal model was defined as: glmer (Error Rate ~ Condition × Word Status + List + (1|Subject) + (1|Item), family = binomial). LRTs confirmed that fit of the model significantly improved when the fixed effect of Condition was included (*LRT*: χ^2^(3) = 37.36, *p* < .001). However, the model fit was not improved by including the fixed effects of Word Status (*LRT*: χ^2^(1) = 3.10, *p* = .078), List (*LRT*: χ^2^(3) = 4.21, *p* = .240), or the interaction term Condition × Word Status (*LRT*: χ^2^(3) = 2.08, *p* = .556). This result suggests that there were no main effects of Word Status or List. Therefore, the optimal model was glmer (Error Rate ~ Condition + (1|Subject) + (1|Item), family = binomial). As with the RT data, the intercept represents performance in the ASD word condition; all other estimates are relative to this value. All conditions had lower error rates compared with the ASD condition (Intercept: β = −2.87, *OR*: 0.06, *SE* = 0.14, *Z* = −20.35, *p* < .001, Identity: β = −0.85, *OR* *=* 0.43 *SE* = 0.17, *Z* = −5.08, *p* < .001, 1SYL_SAME: β = −0.85, *OR* = 0.43, *SE* = 0.17, *Z* = −5.10, *p* < .001, TS: β = −0.35, *OR* = 0.70, *SE* = 0.15, *Z* = −2.44, *p* < .05).

### Discussion

Experiment 2 revealed that priming effects can be obtained in “same” judgements of the same–different task when Hangul primes and targets share a single syllable block in the same position, or when primes and targets share both syllable blocks but in different positions. These results depart from the results reported in Rastle et al. (2019, Experiment 5). Using similar stimulus conditions in a lexical decision task, those authors reported null masked priming effects when disyllabic primes and targets shared only a single syllable block in the same position or shared both syllable blocks in different positions.

These results are consistent with those observed in Experiment 1, and by Kinoshita et al. (2012) and Boudelaa et al. (2019). They would appear to suggest flexible position coding of Hangul syllable blocks at some pre-lexical stage of processing. However, once again, we observed a significant effect of lexical status on “same” judgements, which appears difficult to reconcile with an account of the same–different task as reflecting pre-lexical orthographic representations (Kinoshita et al., 2012; Norris & Kinoshita, 2009). We consider this finding in the “General Discussion” section.

## General discussion

Substantial research has sought to understand the nature of the orthographic representations that support skilled reading. One of the major insights of this research is that information about letter position does not appear to be absolute: skilled readers perceive transposed-letter stimuli as similar to their base words (Perea & Lupker, 2003; Schoonbaert & Grainger, 2004). The wide body of data using transposed-letter phenomena to diagnose the representation of position information has led to a number of competing theories of orthographic processing, all of which put forward hypotheses as to why there is perceptual uncertainty of letter position.

The claim based originally on Hebrew data that this perceptual uncertainty might be language specific is very important theoretically (Frost, 2012). For one, it would mean that perceptual uncertainty of letter position is not caused by low-level visual (e.g., Grainger et al., 2016) or neurobiological factors (Dehaene et al., 2005). It would also indicate that relatively high-level structural or statistical properties of writing systems can constrain learned representations at a much lower level. The theoretical implications would be different if it turned out that position was coded flexibly in orthographic representations across writing systems, and that differences across writing systems reflect how one uses those representations in word recognition tasks (Norris & Kinoshita, 2012a).

The two experiments presented here sought to contribute to this debate by assessing the circumstances in which transposed letter and syllable block priming effects arise in Korean Hangul reading. Previously, we had demonstrated that these effects are not observed in masked priming of lexical decision in Hangul, despite observing identity priming effects of typical magnitude (Rastle et al., 2019). These experiments tested whether this same pattern of priming effects would be observed on “same” judgements in the same–different task, a task that has been argued to reflect pre-lexical orthographic representations (Kinoshita et al., 2012). The results of the priming manipulations were straightforward. In contrast to the null effects documented by Rastle et al. (2019), we observed highly significant priming in both cases on “same” judgements in the same–different task, using very similar stimuli as in our earlier study.

## Task effects on transposition priming in Hangul

These results add weight to the claims of Kinoshita et al. (2012) and Boudelaa et al. (2019) regarding the apparent rigidity of position coding in Semitic writing systems. Our results suggest that like these Semitic writing systems, masked transposition priming effects arise in Hangul when the task involves matching to a previously-shown referent, but not when it involves making a lexical decision. Our findings are important because they demonstrate that this pattern of results is not restricted to writing systems with the tri-consonantal root structure.

We believe that this pattern of results for Hangul word processing can be explained using the same logic as previously articulated for Semitic writing systems (e.g., Boudelaa et al., 2019; Norris & Kinoshita, 2012a). Specifically, responding in the lexical decision task requires participants to accumulate sufficient evidence to determine whether a printed stimulus is a known word. Word identification in the lexical decision experiments reported by Rastle et al. (2019) required precise position information due to the very high proportion of transposed-letter and transposed-syllable anagrams in disyllabic Hangul words. It is not efficient in these cases for readers to allow printed stimuli to activate multiple possible candidates; doing so would frequently result in misidentifications. It may be that readers diminish the activation of possible candidates through strong inhibitory connections that drive down activation of lexical representations that do not provide an exact match to targets (e.g., Davis & Lupker, 2006). This state of affairs would explain the absence of masked transposition priming (Rastle et al., 2019) and masked form priming (Kim & Davis, 2002) in Hangul lexical decision. In contrast, responding in the same–different task requires participants to accumulate only enough evidence to determine whether the target matches a previously presented reference stimulus. This decision is much less reliant on precise orthographic information given that “different” judgements involve reference stimuli *comprising totally different syllable blocks to targets*. The “same” decision can therefore be made on the basis of *any* overlap between reference and target in their syllable blocks. If the same–different judgement required position information—for example, if “different” trials used reference stimuli that were anagrams of targets—we might predict that evidence of position rigidity would reappear.

Our findings also provide new insight into the absence of transposition effects in lexical tasks in Hangul. Previously, Lee and Taft (2009, 2011) had suggested that the fixed structure within Hangul syllable blocks yielded a situation in which letters could be assigned very rapidly to sub-syllabic positions, thereby leaving no opportunity for letter transposition effects to emerge. Later, Rastle et al. (2019) advanced an explanation based on orthographic density, arguing that the reasoning of Lee and Taft (2009, 2011) provided no explanation for the absence of transposition effects in disyllabic words for whole syllable blocks. Our findings would appear to favour the latter argument: if letters are assigned to sub-syllabic positions very rapidly, and if this is the reason why we do not see transposed letter effects in lexical decision, then we might also have expected an absence of transposition effects in the same–different task. The only way around this would be to argue that the assignment of letters to sub-syllabic slots is too fast to allow a transposition effect to emerge in the lexical decision task, but too slow to prevent one in the same–different task. We find this ad hoc line of reasoning to be less compelling than the argument we have advanced regarding the impact of orthographic density on same–different judgements versus lexical decisions. However, it is important to note that this conclusion does not reduce the importance of the syllable block in the Hangul writing system. Indeed, it is because of the rigid, syllable block structure that the Hangul writing system is characterised by a high degree of orthographic density (Rastle et al., 2019).

Thus far, we have commented only on the transposition effects and why they differ in the same–different and lexical decision tasks. However, our findings also suggest that “same” judgements in the same–different task are facilitated by masked primes sharing a single syllable block in the *same* position, while this is not the case in lexical decision (Rastle et al., 2019). We believe that these findings can be explained in the same way as we have offered for the pattern of transposition effects. That is, because of the rigid syllable block structure of the Hangul writing system, it is very common for disyllabic words to have many syllable neighbours (i.e., words differing by only one syllable). The lexical decision task in Hangul essentially requires discrimination between known combinations of syllable blocks and unknown combinations of the same syllable blocks. Substantial evidence is needed to perform this task accurately; performance is undermined if decisions are based on imprecise information about syllable position or identity. In contrast, the same–different task requires a judgement of whether the target is the same or completely different from a reference stimulus. This means that a decision in the same–different task can be made on the basis of only partial information about the identity and position of target syllable blocks; if *any* syllable occurs in both the reference and the target then the response must be “same.” Once again, it would be interesting to test whether this syllable block priming effect were maintained if the same–different judgement required discrimination against syllable-block neighbours (e.g., if reference stimuli in “different” trials were one syllable block different from targets).

## Effect of lexical status in the same–different task

Despite the fit of our data to the general account of position flexibility and rigidity offered by Kinoshita et al. (2012) and Boudelaa et al. (2019), our findings seem inconsistent with their claims that the same–different task reflects pre-lexical processing. The same–different task does not *require* lexical processing, and the fact that masked priming effects are observed on “same” judgements for both word and nonword targets has previously been used to claim that the task reflects pre-lexical processing (Kinoshita & Norris, 2009; Norris & Kinoshita, 2008). However, in both of our experiments “same” judgements to words were made significantly more rapidly than those to nonwords. We doubt that this finding is due to an idiosyncrasy of our items, as a similar effect has been observed in the same–different masked priming experiments reported by Norris and Kinoshita (2008), Kinoshita and Norris (2009), and Kinoshita et al. (2012). These authors focused on the absence of an interaction between lexicality and priming (i.e., that masked priming effects were also observed for nonword targets). However, the presence of a main effect of lexicality is hard to reconcile with the view that the same–different task reflects pre-lexical processes. This conclusion resonates with recent observations of translation priming (Lupker, Perea, & Nakayama, 2015) and phonological priming (Lupker, Nakayama, & Perea, 2015) in this task.

Kinoshita and Norris (2009) discussed this issue very briefly and proposed on the basis of earlier work on visual comparison tasks (Marmurek, 1989) that the word advantage arises due to the ease of *encoding* familiar stimuli. We are not sure what is meant by this argument; for example, whether it refers to the ease of encoding the reference, the target, or both. It may be that it is easier to hold the reference in memory if it is a word. However, irrespective of whether this is the case, we are not sure how this proposal is consistent with an absence of lexical involvement in this task. Indeed, Marmurek (1989) himself argued that the familiarity effect (lexicality effect, in our terminology) “arises from the facilitation in processing due to the activation of a word’s cognitive code” (p. 488) and that “familiarity effects occur when cognitive units for words are activated” (p. 488). The notion of a word’s “cognitive code” seems to us to resonate with that of a lexical representation.

One interesting proposal suggested by a reviewer is that masked priming effects in the same–different task may reflect the impact of the prime on retrieval of the *reference* item as opposed to activation the target. For “same” judgements, the reference item is the same as the target, so any relationship between the prime and the target is also present between the prime and the reference. If the prime refreshes memory of the reference, then this provides a mechanism by which related primes facilitate “same” judgements. For “different” judgements, the reference item is different from the target, so any relationship between the prime and the target will not also be present between the prime and the reference. This means that primes would not be expected to support memory for the reference in “different” trials, and hence no priming would be expected (and none is observed; Kinoshita et al., 2012; Norris & Kinoshita, 2008). We do not believe that this alternative conceptualisation of the task undermines our main result (i.e., that transposed letter and syllable block primes facilitate “same” judgements), because transposed primes are still having a greater impact than substitution primes. However, this alternative conceptualisation does help us to think about the effect of lexical status on “same” judgements, since retrieval of the reference would be expected to be faster in the case of words.

It is important to emphasise that we do not need to demonstrate that the same–different task reflects wholly pre-lexical processing in order for our claims regarding the nature of evidence required in the same–different task versus the lexical decision task to be supported. The same–different task could be influenced by lexical factors (e.g., it could be easier to hold a reference stimulus in mind if it is a word than a nonword), without undermining our claim that a greater degree of precision is required to identify a Hangul stimulus than to determine whether it is the same or completely different to another stimulus. However, it seems clear based on our findings and those of Lupker and colleagues (Lupker, Nakayama, & Perea, 2015; Lupker, Perea, & Nakayama, 2015) that further work is required to understand how participants are performing the same–different task.

## Conclusion

Overall, our results contribute to an emerging picture regarding the flexibility or rigidity of position coding in word recognition. Specifically, they add weight to the proposal that orthographic representations are always characterised by a degree of uncertainty, but that the interrogation of those representations during the recognition process may prioritise precise position information in some writing systems. This emerging picture has so far been driven by findings from Semitic languages with tri-consonantal root morphology (Boudelaa et al., 2019; Kinoshita et al., 2012). The present work together with the work of Rastle et al. (2019) extends this to Korean Hangul, a writing system whose rigid syllabic structure makes precise position information vital for accurate word identification. One important goal for future research will be to home in on a more concrete understanding of the nature of the statistics that make position information vital in some writing systems but not in others, and in some tasks but not in others.

## References

[bibr1-1747021821997336] Bates D. Maechler M. Bolker B. Walker S. (2015). lme4: Linear mixed-effects models using Eigen and S4 (R package version 1.1-10). http://CRAN.R-project.org/package=lme4

[bibr2-1747021821997336] Boudelaa S. Norris D. Mahfoudhi A. Kinoshita S. (2019). Transposed letter priming effects and allographic variation in Arabic: Insights from lexical decision and the same-different task. Journal of Experimental Psychology. Human Perception and Performance, 45, 729–757. 10.1037/xhp0000621 31120301 PMC6532566

[bibr3-1747021821997336] Coltheart M. Rastle K. Perry C. Langdon R. Ziegler J. (2001). DRC: A dual route cascaded model of visual word recognition and reading aloud. Psychological Review, 108(1), 204–256. 10.1037/0033-295x.108.1.204 11212628

[bibr4-1747021821997336] Crepaldi D. Rastle K. Davis C. J. Lupker S. J. (2013). Seeing stems everywhere: Position-independent identification of stem morphemes. Journal of Experimental Psychology: Human Perception and Performance, 39(2), 510–525. 10.1037/a0029713 22905908

[bibr5-1747021821997336] Davis C. J. (2010). The spatial coding model of visual word identification. Psychological Review, 117(3), 713–758. 10.1037/a0019738 20658851

[bibr6-1747021821997336] Davis C. J. Lupker S. J. (2006). Masked inhibitory priming in English: Evidence for lexical inhibition. Journal of Experimental Psychology: Human Perception and Performance, 32(3), 668–687. 10.1037/0096-1523.32.3.668 16822131

[bibr7-1747021821997336] Dehaene S. Cohen L. Sigman M. Vinckier F. (2005). The neural code for written words: A proposal. Trends in Cognitive Sciences, 9(7), 335–341. 10.1016/j.tics.2005.05.004 15951224

[bibr8-1747021821997336] Duñabeitia J. A. Laka I. Perea M. Carreiras M. (2009). Is Milkman a superhero like Batman? Constituent morphological priming in compound words. European Journal of Cognitive Psychology, 21(4), 615–640. 10.1080/09541440802079835

[bibr9-1747021821997336] Forster K. I. Forster J. C. (2003). DMDX: A Windows display program with millisecond accuracy. Behavior Research Methods, Instruments, & Computers, 35(1), 116–124. 10.3758/BF03195503 12723786

[bibr10-1747021821997336] Frost R. (2012). Towards a universal model of reading. Behavioral and Brain Sciences, 35(5), 263–279. 10.1017/S0140525X11001841 PMC3677812 22929057

[bibr11-1747021821997336] Gomez P. Ratcliff R. Perea M. (2008). The overlap model: A model of letter position coding. Psychological Review, 115(3), 577–600. 10.1037/a0012667 18729592 PMC2597794

[bibr12-1747021821997336] Gomez P. Silins S. (2012). Visual word recognition models should also be constrained by knowledge about the visual system. Behavioral and Brain Sciences, 35(5), 287–287. 10.1017/S0140525X12000179 22929059

[bibr13-1747021821997336] Grainger J. Dufau S. Ziegler J. C. (2016). A vision of reading. Trends in Cognitive Sciences, 20(3), 171–179. 10.1016/j.tics.2015.12.008 26809725

[bibr14-1747021821997336] Grainger J. Jacobs A. M. (1996). Orthographic processing in visual word recognition: A multiple read-out model. Psychological Review, 103(3), 518–565. 10.1037/0033-295X.103.3.518 8759046

[bibr15-1747021821997336] Grainger J. Whitney C. (2004). Does the huamn mnid raed wrods as a wlohe? Trends in Cognitive Sciences, 8, 58–59. 10.1016/j.tics.2003.11.006 15588808

[bibr16-1747021821997336] Kim J. Davis C. (2002). Using Korean to investigate phonological priming effects without the influence of orthography. Language and Cognitive Processes, 17(6), 569–591. 10.1080/01690960143000281

[bibr17-1747021821997336] Kinoshita S. Norris D. (2009). Transposed-letter priming of prelexical orthographic representations. Journal of Experimental Psychology: Learning, Memory and Cognition, 35(1), 1–18. 10.1037/a0014277 19210078

[bibr18-1747021821997336] Kinoshita S. Norris D. Siegelman N. (2012). Transposed-letter priming effect in Hebrew in the same–different task. Quarterly Journal of Experimental Psychology, 65(7), 1296–1305. 10.1080/17470218.2012.655749 22494148

[bibr19-1747021821997336] Lally C. Taylor J. S. H. Lee C. H. Rastle K. (2020). Shaping the precision of letter position coding by varying properties of a writing system. Language, Cognition and Neuroscience, 35(3), 374–382. 10.1080/23273798.2019.1663222

[bibr20-1747021821997336] Lee C. H. Kwon Y. Kim K. Rastle K. (2015). Syllable transposition effects in Korean word recognition. Journal of Psycholinguistic Research, 44(3), 309–315. 10.1007/s10936-015-9353-7 25694048

[bibr21-1747021821997336] Lee C. H. Taft M. (2009). Are onsets and codas important in processing letter position? A comparison of TL effects in English and Korean. Journal of Memory and Language, 60(4), 530–542. 10.1016/j.jml.2009.01.002

[bibr22-1747021821997336] Lee C. H. Taft M. (2011). Subsyllabic structure reflected in letter confusability effects in Korean word recognition. Psychonomic Bulletin & Review, 18, 129–134. 10.3758/s13423-010-0028-y 21327354

[bibr23-1747021821997336] Lerner I. Armstrong B. C. Frost R. (2014). What can we learn from learning models about sensitivity to letter-order in visual word recognition? Journal of Memory and Language, 77, 40–58. 10.1016/j.jml.2014.09.002 25431521 PMC4242428

[bibr24-1747021821997336] Lupker S. J. Nakayama M. Perea M. (2015). Is there phonologically based priming in the same−different task? Evidence from Japanese−English bilinguals. Journal of Experimental Psychology: Human Perception and Performance, 41(5), 1281–1299. 10.1037/xhp0000087 26076173

[bibr25-1747021821997336] Lupker S. J. Perea M. Davis C. J. (2008). Transposed-letter effects: Consonants, vowels and letter frequency. Language and Cognitive Processes, 23(1), 93–116. 10.1080/01690960701579714

[bibr26-1747021821997336] Lupker S. J. Perea M. Nakayama M. (2015). Non-cognate translation priming effects in the same–different task: Evidence for the impact of “higher level” information. Language, Cognition and Neuroscience, 30(7), 781–795. 10.1080/23273798.2015.1015430

[bibr27-1747021821997336] Marmurek H. H. (1989). Familiarity effects and word unitization in visual comparison tasks. Memory & Cognition, 17(4), 483–489. 10.3758/BF03202622 2761406

[bibr28-1747021821997336] National Institute of the Korean Language. (2001). Korean word database. 21st-Century Sejong Project Corpus, Seoul, Korea.

[bibr29-1747021821997336] Norris D. Kinoshita S. (2008). Perception as evidence accumulation and Bayesian inference: Insights from masked priming. Journal of Experimental Psychology. General, 137(3), 434–455. 10.1037/a0012799 18729709

[bibr30-1747021821997336] Norris D. Kinoshita S. (2012a). Orthographic processing is universal; it’s what you do with it that’s different. Behavioral and Brain Sciences, 35(5), 296–297. 10.1017/S0140525X12000106 22929590

[bibr31-1747021821997336] Norris D. Kinoshita S. (2012b). Reading through a noisy channel: Why there’s nothing special about the perception of orthography. Psychological Review, 119(3), 517–545. 10.1037/a0028450 22663560

[bibr32-1747021821997336] Perea M. Abu Mallouh R. Carreiras M. (2010). The search for an input-coding scheme: Transposed-letter priming in Arabic. Psychonomic Bulletin & Review, 17(3), 375–380. 10.3758/PBR.17.3.375 20551361

[bibr33-1747021821997336] Perea M. Carreiras M. (2006). Do transposed-letter similarity effects occur at a syllable level? Experimental Psychology, 53(4), 308–315. 10.1027/1618-3169.53.4.308 17176663

[bibr34-1747021821997336] Perea M. Lupker S. J. (2003). Transposed-letter confusability effects in masked form priming. In Kinoshita S. Lupker S. J. (Eds.), Masked priming: State of the art (pp. 97–120). Psychology Press.

[bibr35-1747021821997336] Perea M. Pérez E. (2009). Beyond alphabetic orthographies: The role of form and phonology in transposition effects in Katakana. Language and Cognitive Processes, 24(1), 67–88. 10.1080/01690960802053924

[bibr36-1747021821997336] R Core Team. (2016). R: A language and environment for statistical computing [Computer software]. R Foundation for Statistical Computing, Vienna, Austria. http://www.R-project.org

[bibr37-1747021821997336] Rastle K. Lally C. Lee C. H. (2019). No flexibility in letter position coding in Korean. Journal of Experimental Psychology: Human Perception and Performance, 45(4), 458–473. 10.1037/xhp0000617 30762419

[bibr38-1747021821997336] Schoonbaert S. Grainger J. (2004). Letter position coding in printed word perception: Effects of repeated and transposed letters. Language and Cognitive Processes, 19(3), 333–367. 10.1080/01690960344000198

[bibr39-1747021821997336] Velan H. Frost R. (2007). Cambridge University versus Hebrew University: The impact of letter transposition on reading English and Hebrew. Psychonomic Bulletin & Review, 14(5), 913–918. 10.3758/BF03194121 18087959

[bibr40-1747021821997336] Velan H. Frost R. (2009). Letter-transposition effects are not universal: The impact of transposing letters in Hebrew. Journal of Memory and Language, 61(3), 285–302. 10.1016/j.jml.2009.05.003 20161017 PMC2748959

[bibr41-1747021821997336] Velan H. Frost R. (2011). Words with and without internal structure: What determines the nature of orthographic and morphological processing? Cognition, 118(2), 141–156. 10.1016/j.cognition.2010.11.013 21163472 PMC3027305

[bibr42-1747021821997336] Witzel N. Qiao X. Forster K. (2011). Transposed letter priming with horizontal and vertical text in Japanese and English readers. Journal of Experimental Psychology: Human Perception and Performance, 37(3), 914–920. 10.1037/a0022194 21639675

